# Welcome to *Perioperative Medicine*

**DOI:** 10.1186/2047-0525-1-1

**Published:** 2012-06-27

**Authors:** Mark Hamilton, Monty G Mythen

**Affiliations:** 1 St. George's Hospital, Blackshaw Road, London, SW17 8QT, UK; 2 University College Hospital, 1st Floor, Maple House, 149 Tottenham Court Road, London, W1T 7DN, UK

## 

We are proud to welcome you to the launch issue of *Perioperative Medicine*. Many before us have campaigned for improvements in the care of patients undergoing surgery and for us this is a major milestone in our clinical and academic careers. As a group we have drawn much inspiration and leadership from Professor David Bennett and have done the majority of our research and teaching on perioperative medicine with the explicit aim of improving the outcome of patients undergoing major surgery [1]. With David and colleagues we have campaigned and lobbied for improved and consistent standards of care [2,3]. We started the Evidence Based Perioperative Medicine International Congress (EBPOM) over a decade ago and now work closely with sister organizations around the world [4,5]. Perioperative medicine is now rightly recognized as a legitimate and independent sub-specialty but the journey is only just beginning [6-8].

The recent publication from the UK’s National Confidential Enquiry into Patient Outcome and Death entitled ‘Peri-operative Care: Knowing the Risk (2011)’ paints a grim picture [9]. It concludes that overall the care of patients was substandard in the majority of high-risk patients. The thirty day mortality in those patients in whom the advisors considered there to have been inadequate pre-operative fluid management was 20.5% compared to 4.7% mortality in those with adequate pre-operative fluid therapy. Patients who suffered intra-operative complications had a thirty day mortality of 13.2% compared to 5.7% in those without. Yet, cardiac output monitoring was rarely used in high-risk patients and inadequate intra-operative monitoring was associated with a three-fold increase in mortality. Reports from the USA tell similar stories. For example, the Veterans’ Association National Surgical Quality Improvement Program (NSQIP) found that the occurrence of a thirty day postoperative complication is the single most important factor determining survival after major surgery [10]. More recently Birkmeyer *et al.* demonstrated that the costs of inpatient surgery are thousands of dollars more per patient on average at hospitals with high complications. They concluded that their “findings suggest that local, regional, and national efforts aimed at improving surgical quality may ultimately reduce costs and improve outcomes” [11]. They went on to note that “achieving superior outcomes in surgery may require that hospitals invest in expensive resources, such as intensivist-staffed intensive care units, high nurse-to-bed ratios, advanced technology, and specialist services”. If improvements are to be made then we must share, spread and adopt best practice whilst examining the efficacy and effectiveness of innovative care pathways and technologies. This is a core objective of the new journal, *Perioperative Medicine*.

In the UK we have recently seen dramatic improvements in the care of patients undergoing elective major surgery as a result of a government-funded Enhanced Recovery Partnership Programme [12]. This is an exemplar of the power of the spread and adoption of best evidence practices through education informed by research. The recently published report demonstrates improved quality of care and cost effectiveness whilst promoting and recognizing investment in innovation [12]. We are delighted to be publishing a Consensus Statement on Perioperative Fluid Management in Enhanced Recovery by the Clinical Leaders of the English Enhanced Recovery Partnership in this, our first issue of *Perioperative Medicine*.

Care of the surgical patient is increasingly recognised to rely on a coordinated approach to healthcare by a multidisciplinary team. *Perioperative Medicine* brings together the best research from all of these fields and applies it to the care of patients undergoing surgery to help healthcare providers improve their patients' outcomes. With the change in population demographics post-operative morbidity has been recognised as one of the epidemics of the 21^st^ century. High quality cost effective changes in practice demand a robust evidence base.

*Perioperative Medicine* is an open access peer-reviewed journal that publishes highly topical clinical research relating to the perioperative care of surgical patients. Its essence is the distillation, examination and application of clinical evidence to improve surgical outcome. Modern perioperative medicine is a true multidisciplinary specialty and the journal welcomes research in all areas relevant to perioperative medicine from any healthcare professional. You will see from our editorial board that we are truly international with members from every continent.

This first issue of *Perioperative Medicine* exemplifies our mission to look across the continuum of care from around the world. It includes articles on pre-operative assessment from China (Lee *et al.*), emergency pain relief for hip fracture patients from Ireland (Szucs *et al.*), quality improvement strategies (Speck *et al.*) and renal failure (Calvert and Shaw) from the USA and peri-operative fluid management from England (Mythen *et al.*).

We hope you enjoy *Perioperative Medicine* and hope that you will engage with comment and submissions.
